# High-Throughput *In-Operando* Terahertz Time-Domain Spectroscopy of Chemical Vapor Deposited Graphene for pH Sensing

**DOI:** 10.1021/acsnano.6c03948

**Published:** 2026-10-21

**Authors:** Oliver J. Burton, Sebastian Engelbrecht, Thomas Goepfert, Ibtisam Hammad Abbasi, Linyuan Xin, Bernd M. Fischer, Hannah Joyce, Stephan Hofmann, Hungyen Lin

**Affiliations:** † Department of Engineering, 2152 University of Cambridge, Cambridge CB3 0FA, United Kingdom; ‡ French-German Research Institute of Saint-Louis, 5 Rue du Général Cassagnou, 68301 Saint-Louis Cedex, France; § School of Engineering, University of Warwick, Coventry CV4 7AL, United Kingdom

**Keywords:** terahertz spectroscopy, CVD graphene, pH sensing, contactless conductivity mapping, in-operando liquid sensing, Raman mapping, defect engineering

## Abstract

Reliable pH sensing in liquid environments underpins applications in energy storage, biosensing, and process monitoring, yet conventional graphene-based electrical devices introduce ohmic contacts, encapsulation layers, and electrochemical artifacts that can obscure intrinsic behavior. These approaches also fail to capture the strong spatial variability of commercial CVD graphene. Here, we present a fully contactless methodology for quantifying pH-dependent conductivity of large-area CVD graphene using through-substrate reflection terahertz time-domain spectroscopy (THz-TDS). A customized liquid cell with a THz transparent window preserves unrestricted access to the graphene–electrolyte interface while enabling centimeter-scale conductivity mapping and *in-operando* single-point monitoring during sequential pH modulation. By integrating spatially registered Raman mapping with automated quality-metric filtering, we extract statistically robust trends across thousands of pixels rather than isolated device regions. We show that pristine monolayer graphene exhibits the highest baseline conductivity and strongest pH sensitivity, whereas O_2_-plasma-introduced defects systematically suppress both. This scalable, remote, and inert probing approach establishes THz-TDS as a powerful platform for high-throughput characterization and robust liquid-phase sensing in thin films and 2D systems.

## Graphical abstract

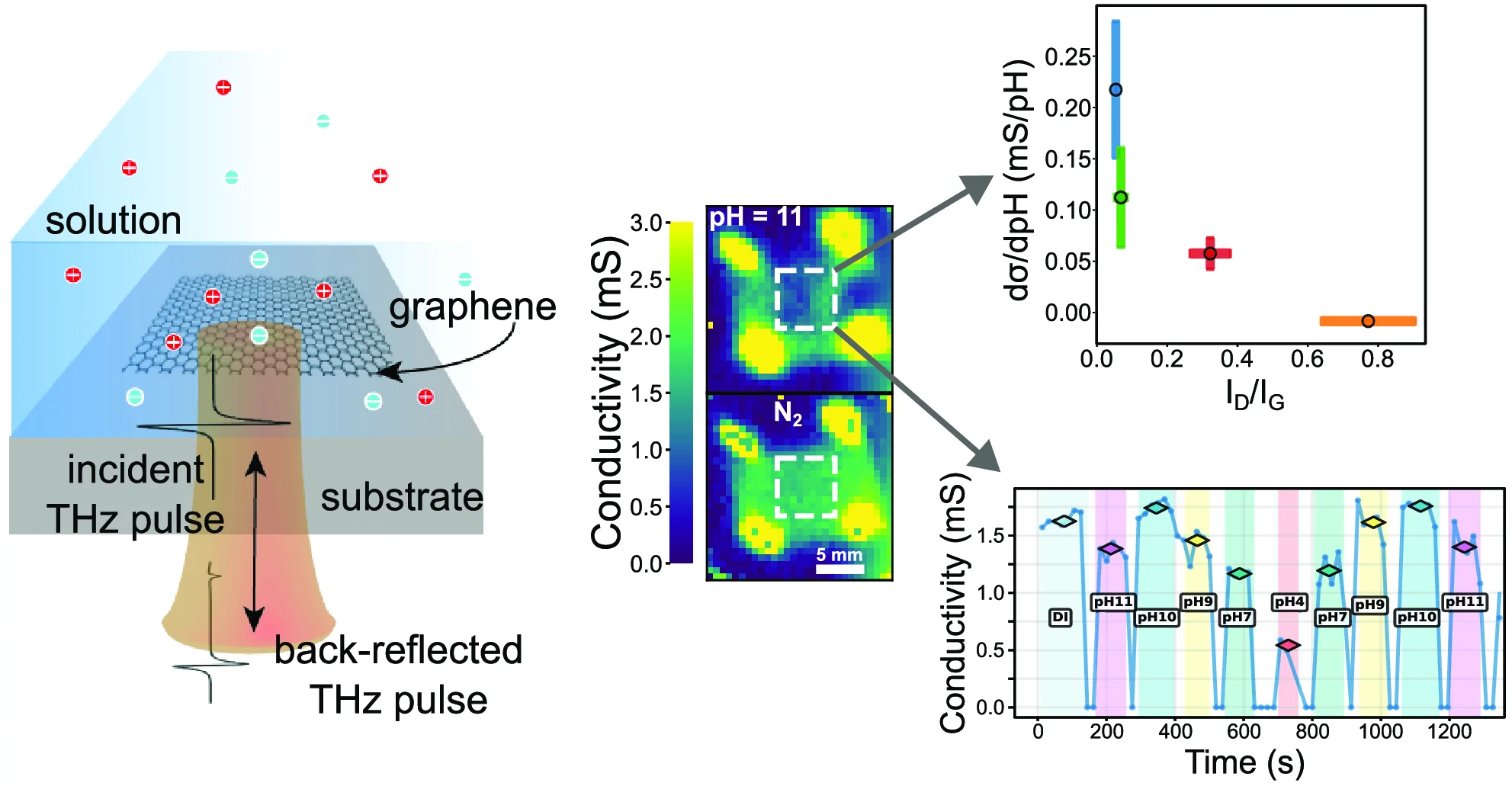

## Introduction

Two-dimensional (2D) materials such as graphene combine exceptional electrical, mechanical, and optical properties that have driven major advances in sensing, energy storage, and nanoelectronics.^1–5^ For liquid-phase chemical sensing, platforms must be both chemically inert across diverse conditions and continuously responsive to subtle compositional changes, such as pH. Graphene’s high carrier mobility, large surface-to-volume ratio, chemical stability, and atomic thinness make it an attractive candidate for these applications, including pH and biomolecular sensing in electrolyte-gated geometries.^6–10^ However, any viable pH sensing platform must not only exhibit high sensitivity, but also remain inert to its environment, avoiding perturbation of local ionic composition, or flow fields during operation.

This requirement is particularly challenging for CVD graphene, where the same sensitivity that enables detection also amplifies the impact of fabrication residues, ambient adsorbates, and growth-induced inhomogeneities. Even small variations in polymer or etchant residues, atmospheric doping, or encapsulation chemistry can significantly alter carrier density and mobility.^11–19^ Commercial CVD films exhibit pronounced spatial heterogeneity in continuity, grain structure, and electrical properties, meaning that local measurements at individual device level and of select regions may not be representative of the wafer-scale behavior.^20–22^

Electrical and electrochemical pH sensors, including glass electrodes, ion-sensitive field-effect transistors, and graphene electrolyte-gated devices, are well-established and can provide sensitive, calibrated pH readout for many applications. However, when the objective is to remotely interrogate the response of material/liquid interfaces, contacted device architectures introduce additional interfaces and sources of variability. Lithographically patterned contacts, local metal work functions, and contact geometries can unintentionally dope or damage the graphene and give rise to spatially varying contact resistance and hysteresis.^23–26^ In liquid environments, contacts and passivation layers further influence the local ionic environment near the sensing interface, impose electrochemical constraints, and limit compatibility with aggressive or complex media unless carefully encapsulated and integrated with counter and reference electrodes.^6,7,9,21,27,28^ Crucially, measurements will be restricted to a limited number of “hero” devices, making it difficult to disentangle intrinsic pH-dependent responses from local variability, environmental drift, or device-to-device scatter, further exacerbated by the strong spatial heterogeneity of graphene at the wafer scale.^29–32^

Optical approaches such as Raman spectroscopy offer noninvasive access to strain, doping, and defect metrics, and have been extensively used to infer graphene’s electrical properties via correlations between *I*_D_/*I*_G_ ratios, 2D peak widths, and carrier mobility.^33–36^ However, conductivity estimates remain indirect and empirical, where defect engineering, such as by oxygen plasma (as in this work) or related treatments, further complicates interpretation.^37–41^

In this context, terahertz time-domain spectroscopy (THz-TDS) provides a powerful contactless probe of graphene’s Drude conductivity, enabling spatially resolved mapping of sheet conductance and mobility without any lithographic metallisation.^20,42–51^ Our previous work established the feasibility of through-substrate reflection THz-TDS,^50^ but did not provide local environment control. Here, we demonstrate the first contactless, *in-operando* mapping of CVD graphene conductivity under liquid pH modulation using through-substrate reflection THz-TDS combined with a customized liquid cell, schematically illustrated in Figure 1. By illuminating the sample through the supporting substrate, we preserve the previously established contactless spatial mapping capabilities while providing unobstructed access to the graphene–liquid interface, in a versatile configuration that is inherently inert with respect to the probed solution and compatible with strongly absorbing aqueous media.^52–54^ This enables dynamic pH control and continuous monitoring of graphene’s DC conductivity without electrodes, passivation layers, or local electrochemical perturbations. Crucially, the ability to premap conductivity over centimeter-scale areas and then select optimal sensing regions is effectively equivalent to screening hundreds of individual devices in a single experiment.^20,42^ To fully exploit this high-throughput capability, we implement an automated THz–Raman analysis framework that performs image registration, quality-metric-based pixel rejection, and statistical aggregation over thousands of measurement points per sample. This multimodal data processing approach allows us to extract statistically relevant trends, such as distributions of dσ/dpH across realistic CVD films, as opposed to isolated/arbitrarily selected regions, and to incorporate intrinsic pH-dependent trends from growth, transfer, and defect-induced inhomogeneities into the sample measurement. By comparing statistically meaningful THz conductivity maps with spatially registered Raman metrics across pristine and deliberately defect-engineered samples, we show that transferred pristine CVD graphene exhibits superior pH sensitivity and conductivity than its defective counterparts. This behavior contrasts with expectations derived from reduced graphene oxide based pH sensors, where increasing defect density is often associated with enhanced electrochemical response.^41^ This divergence highlights the nontrivial interplay between defects, residues, structural corrugations, and interfacial doping in governing the liquid-phase response of large-area transferred CVD graphene films for robust, inert, and spatially reliable pH sensing.

**Figure 1:**
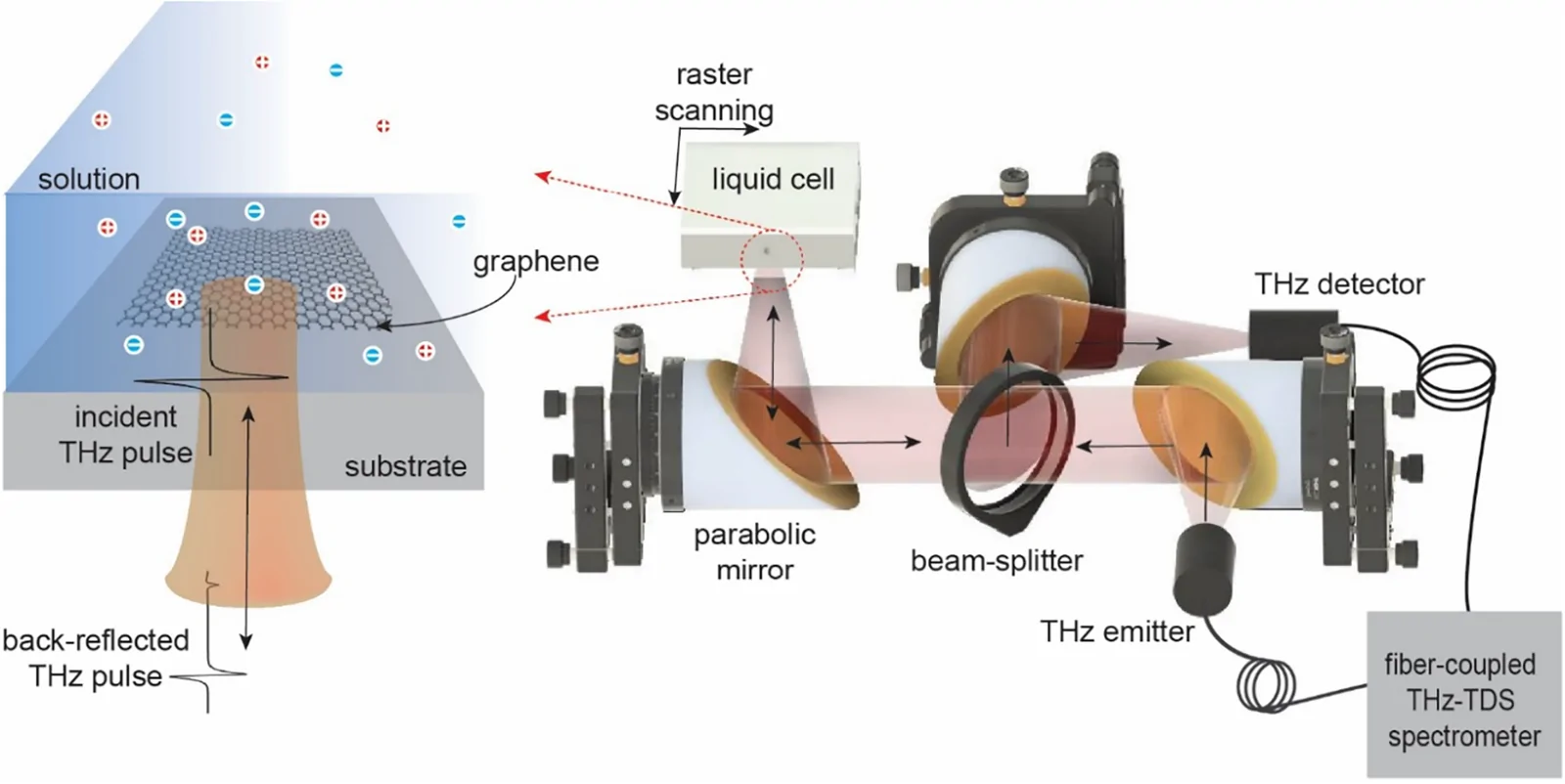
Generated THz pulses are focused by a parabolic mirror into a customized liquid cell with a THz transparent viewing window that also functions as graphene support enabling measurements in aqueous solutions. Back-reflected THz pulses are detected by a THz photoconductive antenna via THz guiding optics.

## Results/Discussion

We characterize and map graphene’s THz response in nitrogen atmosphere, and in 5 different pH buffer solutions to probe the response and mechanism behind the pH dependent response of isolated CVD graphene in aqueous environments. Figure 2 shows a representative plot of Drude DC conductivity from one sample (sample B) across 5 different pH conditions (a trend consistent across other samples, see SI Figure S5). The mean conductivity increases with increasing pH, consistent with prior reports on electrical-contact-based devices.^6,7,41^ It should be noted that if these contacts to graphene are not fully encapsulated, they will affect device response.^25^ Despite relatively high standard deviation, the conductivity trend is consistently observed across all pixels, with a monotonic rate of change relative to pH. This variability reflects the innate spatial heterogeneity of transferred CVD graphene arising from imperfections introduced during growth and transfer,^17,18,20,24^ and is consistent with the variability reported for CVD graphene based devices.^21,22^ Despite local variability in electronic qualities, the trend presented here is in agreement with previous work on graphene based pH sensors.^39,41^

**Figure 2:**
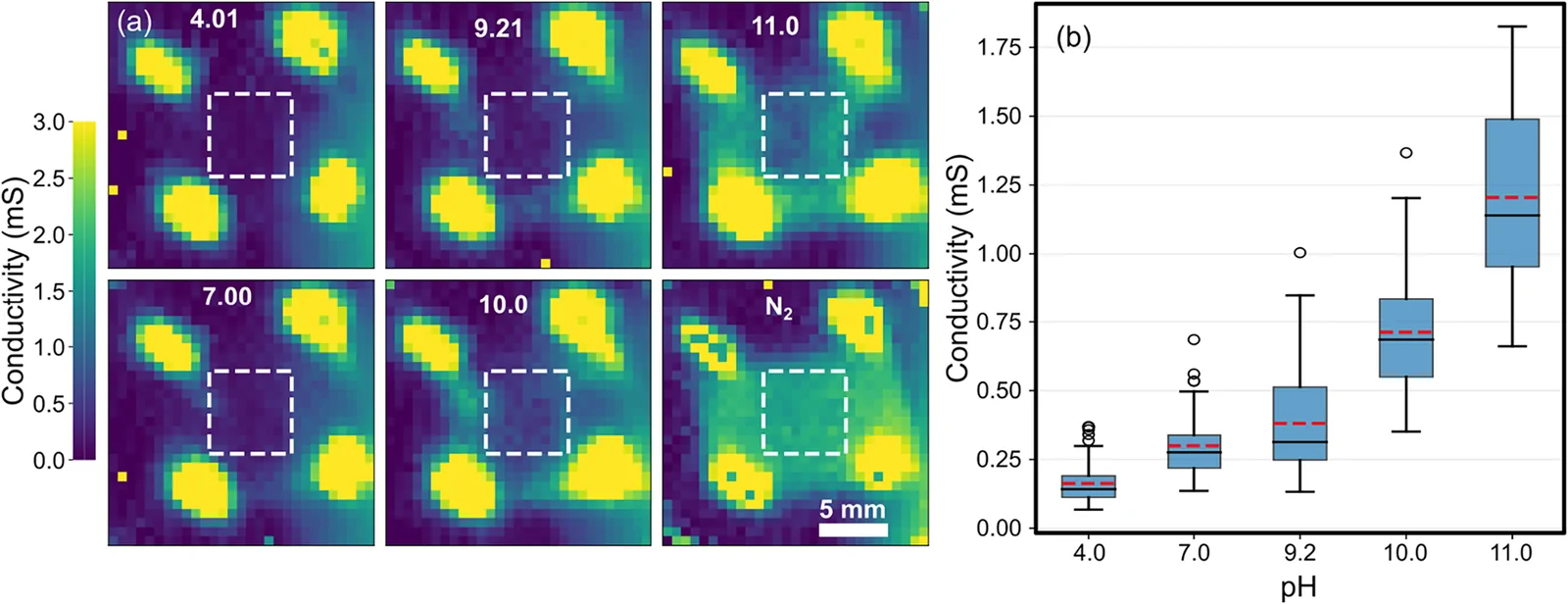
(a) The individual Drude DC conductivity maps for the data shown in (b), as well as the N_2_ measurement for a representative sample (B) prior to measurement. The white boundaries indicate the regions of interest from which the data to produce (b) was acquired and is placed within the 4 Au alignment markers (seen here as the bright yellow spots). (b) A box-plot series showing the statistics of Drude DC conductivities of graphene (sample B) with respect to pH, extracted from valid data points over the ∼1 × 1 cm^2^ region shown in (a). The red dashed line shows the mean value, the black lines in the box show the 25th, 50th and 75th quartiles, the whiskers show the range within 1.5× the interquartile range and the circles denote points outside this range. Note that the box-plot spread represents spatial pixel-to-pixel variability across the mapped graphene region, not repeated-measurement uncertainty at a single point.

Motivated by this strong response of the Drude DC conductivity to pH, shown in Figure 2, and its dependence on defect levels in graphene (SI Figure S3 as well as literature on r-GO),^41^ we investigate how defect density influences the pH sensitivity of CVD graphene. Five samples (A–E) were prepared, with samples A–D used for mapping and sample E used for *in-operando* demonstrations. All samples consist of graphene grown using previously reported methods^16^ and wet-transferred onto float-zone Si substrates. To modulate defect density, selected samples were exposed to controlled O_2_reactive-ion etching (RIE).^37^ Literature suggests that this treatment can introduce a combination of point/vacancy-type and sp^3^-type defects, with more extreme O_2_ plasma treatment potentially leading to structural discontinuities.^38–40^ Graphene defect density was quantified using the Raman D-to-G peak intensity ratio (*I*_D_/*I*_G_), an established metric empirically relating to the defect density in graphene films.^33,37^ Defects were introduced by iterative O2 RIE exposure, using 10 s plasma steps followed by Raman mapping after each step.^19^ Rather than using nominal plasma exposure time as the defect metric, we used the mapped Raman *I*_D_/*I*_G_ ratio measured after treatment to quantify the final defect density, since the effect of plasma exposure can vary spatially across transferred CVD graphene as well as between as-transferred samples. The treatment was stopped once the mapped mean *I*_D_/*I*_G_ ratio approached the desired target range for each sample. Figure 3 shows this mapping process, with exemplar Raman maps of the *I*_D_/*I*_G_ ratios of pristine and highly defective graphene. Resulting *I*_D_/*I*_G_ ratios are shown in Figure 3(c) for the different samples, and span means between <0.05 to 0.76. Figure 3 also reveals the spatial distribution of defect densities, which directly influences THz conductivity measured under N_2_ atmosphere prior to liquid immersion. Each point corresponds to the mean conductivity (or rate of change of mean conductivity) calculated across all spatial registered, valid pixels, with error bars indicating the standard deviation of the data used to create mean value shown. We observe that increasing defect density correlates to a systematic reduction in Drude DC conductivity.

**Figure 3:**
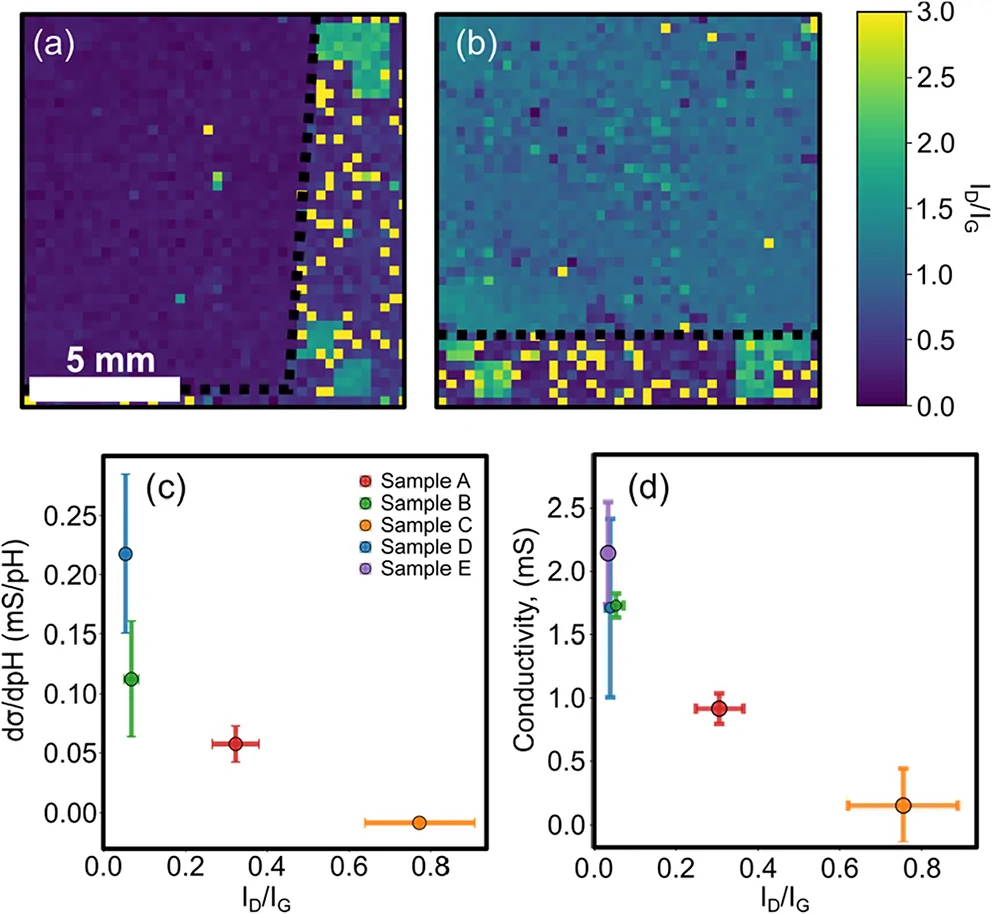
(a) Pristine Raman *I*_D_/*I*_G_ map of sample B. (b) Defective Raman D to G ratio map of sample C. (c) The mean dσ/dpH against mean D to G ratio for all samples measured. (d) The mean THz conductivity measured in N_2_ prior to aqueous measurements against the mean *I*_D_/*I*_G_ ratio for all samples. The dashed lines in (a, b) show the left/upper region where graphene is present.

Although prior studies on defect mediated sensing reported enhanced sensitivity in both pH and gas detection, these observations were largely limited to reduced graphene oxide (r-GO) for electronic pH sensing and to CVD graphene operating in gas-phase environments at low/uncontrolled defect densities.^39,41^ In contrast, here, we investigate a monolayer CVD graphene system using a fully contactless THz approach and introduce controlled defect densities, revealing that defects predominantly suppress the overall conductivity range associated with pH variation, thereby outweighing any potential sensitivity gains from the surface modification resulting from defect introduction. Contrary to expectations from r-GO pH sensors and defect assisted gas sensing in CVD graphene, the highest quality material, characterized by the lowest *I*_D_/*I*_G_ peak ratio from Raman spectroscopy, exhibits both the highest conductivity and the greatest pH sensitivity (Figure 3c) as defined by the mean dσ/dpH, corresponding to the slope obtained from a linear fit of the mean conductivity values versus pH. The sensitivity is maximized in pristine transferred films (*I*_D_/*I*_G_ < 0.05), whereas increasing defect density systematically suppresses the pH dependent conductivity modulation. These findings underscore the importance of using pristine low defect density in CVD graphene for optimal performance in pH sensing applications.

In practical sensing applications, repeatability is a critical performance metric. To demonstrate this, we developed a rudimentary *in-operando* sensing platform where different pH solutions can be added and removed during real-time THz measurement. Figure 4(a) shows the time-resolved conductivity at every ∼1 s on a single-point of a pristine, high conductivity region of sample E, identified from an initial coarse conductivity scan. The temporal traces capture real-time conductivity modulation in response to sequential pH changes, highlighting both the exemplary sensitivity (dσ/dpH ≈ 0.36 mS/pH) and stability (repeatability error of 6 to 9% of full-scale response) of the proposed technique. A consistent and repeatable trend is observed with varying pH, in agreement with the averaged behavior for pristine sample B as shown in Figure 2. To assess repeatability, Figure 4b shows the statistical distribution of ten repeated measurements over the course of an hour revealing minimal deviation across individual cycles. Furthermore, a hysteresis test was performed by cycling the pH from 11 to 4 and back to 11 (Figure 4c). The resulting conductivity profile shows minimal hysteresis under these conditions between the down-sweep and the up-sweep, with a reproducible dip appearing at pH 11, consistent with the plateau behavior observed in spatial maps (SI Figure S5). These results suggest that a careful selection of graphene regions is essential to maximize the range of pH sensing while preserving a monotonic behavior. Within the pH range of 4 to 10, the system demonstrates repeatability, highlighting the potential for use in liquid sensing applications. It should be noted that the temporal resolution of the measurement is ultimately limited by system noise, which can be reduced by increasing the number of waveform averages. Although the averaging used here yields a relatively long acquisition time (see SI), the number of averages can, in principle, be substantially reduced enabling subsecond time resolutions for capturing fast kinetics.

**Figure 4:**
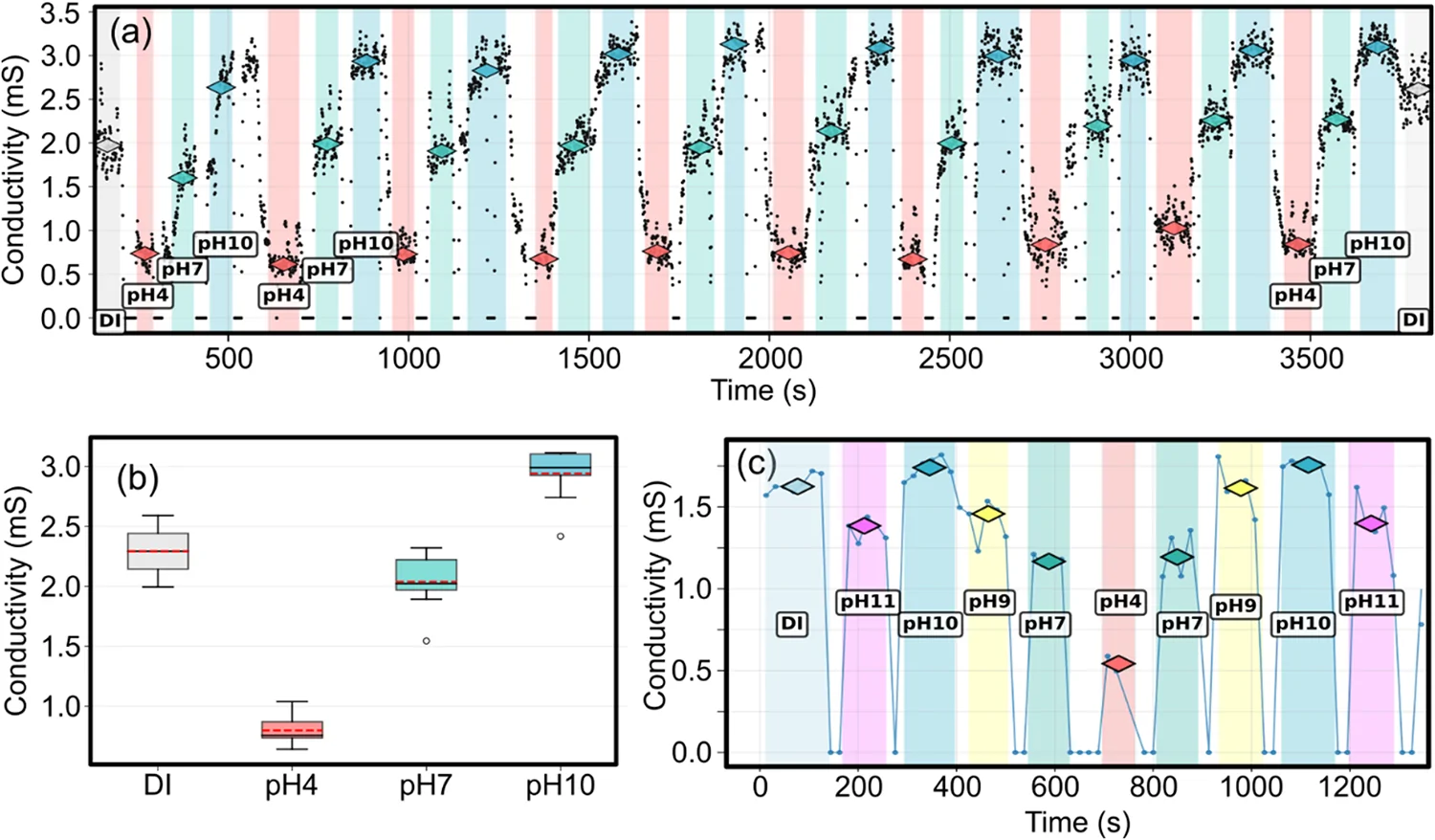
(a) A plot of Drude DC conductivity against time, showing the response to 3 different pH solutions (4, 7 and 10) with 10 repeated measurements spanning the course of an hour. Diamond markers show the mean position of the measurement. (b) A box-plot series showing the statistics of Drude DC conductivities of the graphene region from (a) with respect to pH over the measurement series. The red dashed line shows the mean value, the black lines in the box show the 25th, 50th and 75th quartiles, the whiskers show the range within 1.5× the interquartile range and the circles denote points outside this range. (c) The conductivity against time to illustrate the minimal hysteresis of this particular measurement area, cycling from pH 11 to 4 then back to 11 again.

Prior works on electronic devices have reported a shift in the conductance-gate voltage toward more positive voltages, indicating increased p-doping in graphene.^6–8,27^ In systems without an applied gate voltage, such as the one used here, enhanced p-doping is expected to increase the conductivity. This also implies that measured conductivity should taper-off at higher p-doping levels, which is observed in some of our samples at pHs of 10/11 (see SI Figure S5). Lei et al. previously attributed the sensing mechanism of their two-terminal graphene device to nonfaradaic adsorption of ions with H_3_O^+^ and OH^–^ ions inducing n- and p-doping at low and high pHs, respectively. This is also supported by literature that suggests that cations electrostatically counteract the reported p-doping of CVD graphene by SiO_2_,^6,28^ which is also likely to be present in our system. Extracted carrier densities from THz spectroscopy further shows that carrier density generally increases with increasing pH (see SI Figure S4), thus suggesting that our graphene samples were already sufficiently p-doped to begin with (else an initial decrease in conductivity and carrier density at low pHs would have been observed).

The electric-double-layer model provides another framework for explaining the observed sensing trends. If the transferred graphene is initially p-doped, as suggested by previous reports for CVD graphene on oxidized substrates^13,26,29,32^ and by the Raman-derived doping analysis in Figure S4, then changes in interfacial ion distributions could modulate the graphene Fermi level through an electric-double-layer-like response. In this picture, increasing pH would alter the local balance of H_3_O^+^, OH^–^, and buffer ions near the graphene/liquid interface, contributing to the observed increase in conductivity. As the pH of the solution increases, there is an increased density of OH^–^ groups adjacent to the graphene leading to an increased interfacial potential drop and a decrease in the Fermi-level of the graphene, *i.e*. becoming increasingly p-doped. In alkaline media, reduced proton concentrations and the adsorption of hydroxide or deprotonated surface groups generate a net positive interfacial potential, evidenced by work on anion/cation selection in graphene nanopores.^10^ The mechanistic explanation becomes increasingly complex when considering additional aspects of the system, *e.g*. native oxide in Si, the nature and effect of native defects, tears, grain boundaries, folds or ripples, all present on CVD graphene. SiO_2_, for example, has been found to significantly shift the Dirac point of graphene owing to OH^–^ groups on the surface in air, and may well have similar effects when in aqueous solutions.^6,15,28^ However, the effects of these in the context of sensing mechanisms are yet to be sufficiently understood thus significantly more work in these complicated environments are required to disentangle these processes in CVD graphene in aqueous environments.

The responses observed in this work are likely to arise from an interplay of multiple mechanisms operating across different length scales. Although the THz measurements directly demonstrate a reproducible pH-dependent modulation of graphene conductivity, they do not, by themselves, uniquely identify the microscopic origin of this response. It should also be noted that the commercial buffer solutions used in this study differ not only in pH but also in chemical composition. Therefore, while the monotonic conductivity trend is consistent with pH-dependent interfacial doping, minor contributions from ionic strength, buffer identity, and ion-specific screening effects cannot be excluded.^55^ Accordingly, we interpret the observed trends in terms of several plausible and potentially concurrent mechanisms, including interfacial doping, electric-double-layer formation, ion adsorption and screening, substrate-related effects, and the intrinsic spatial heterogeneity of transferred CVD graphene.

It is important to distinguish the present proof-of-concept platform from established commercial pH sensors. Conventional glass-electrode pH meters and industrial process probes provide calibrated absolute pH readout, often with accuracies on the order of 0.01 pH units, although their response times are typically on the scale of several seconds to minutes depending on probe design, sample conditions, and calibration protocol. In contrast, the value of the proposed THz/graphene approach demonstrated here lies not in competing directly with mature electrochemical pH probes in terms of absolute accuracy, but in enabling contactless interrogation of a conductive sensing interface through a THz-transparent substrate. This geometry eliminates the need for lithographically defined electrical contacts, passivation layers, or immersed electrodes, while also allowing spatially resolved, *in situ* mapping of interfacial response. As such, the platform offers the distinct functionality of monitoring pH-dependent interfacial changes remotely and potentially with high temporal resolution, thus providing complementary capabilities that are not readily accessible with conventional point-contact pH sensors.

Irrespective of the underpinning mechanisms, this study has shown a consistent and repeatable pH modulation of graphene’s conductivity, measured entirely using our proposed approach. This establishes a significant step toward high-throughput, *in-operando* THz measurement of 2D interfaces inferring environmental properties, exemplified here through pH sensing. Building on earlier demonstrations of THz conductivity mapping of graphene in ambient and through-substrate configurations, our liquid-cell implementation confirms that, provided an optically polished THz-compatible substrate is used, sufficient spatial resolutions can be achieved in aqueous environments to enable effective sensing functionalities. This resolution could, in principle, be improved by an order of magnitude through tighter focusing/near-field techniques but this would come at the expense of a significantly longer mapping time.^51^ A key advantage of this methodology is that the THz interaction occurs exclusively with the conductive film, allowing straightforward adaptations to bilayer or multilayer graphene, other 2D materials, and chemically functionalized or hybrid films to tailor specificity toward particular ionic species or analytes. Moreover, the contactless reflection geometry is inherently suited for remote and continuous monitoring, where the acquisition parameters can, in principle, be adjusted to achieve subsecond temporal resolution to access rapid interfacial kinetics. Even higher data acquisition rates may be possible using alternative THz architectures such as asynchronous optical sampling,^56^ which can offer up to kHz waveform acquisitions. Such capabilities make the approach particularly attractive for environments where wired electrodes or conventional device architectures are impractical, including chemically aggressive media or high-throughput continuous production industrial lines. In such scenarios, a single THz-transparent window coated with graphene (or another suitable 2D conductor) could be integrated into a pipe or reactor wall to monitor target environmental parameters in real-time with minimal disruption. This methodology demonstrates a versatile graphene-based metrological platform, and represents the potential broader materials platform for scalable, remote interrogation of liquid–solid interfaces.

## Conclusions

We have demonstrated a contactless, through-substrate THz-TDS approach for quantifying the pH-dependent electrical response of large-area CVD graphene in aqueous environments. By combining wafer-scale THz conductivity mapping with spatially registered Raman *I*_D_/*I*_G_ analysis, we showed that pristine (low-defect) graphene exhibits both the highest baseline conductivity and the strongest pH sensitivity, whereas higher defect density films systematically suppress the responses. *In-operando* single-spot measurements further confirm a repeatable, monotonic conductivity increase over the tested pH range from approximately 4 to 10, with limited hysteresis under the present buffer-exchange conditions. While the platform is, in this work, not calibrated for absolute pH determination, these results establish reflection THz-TDS as a scalable, contactless approach for monitoring liquid-induced conductivity changes in graphene and other 2D material interfaces. Crucially, the through-substrate geometry preserves unrestricted access to the graphene-liquid interface without electrodes or encapsulation, enabling rapid prescreening of optimal regions and mitigating the spatial variability inherent to transferred CVD graphene. Collectively, these results establish reflection THz-TDS as a scalable, noninvasive platform for *in situ* characterization of and *in-operando* remote liquid-phase sensing with 2D materials, motivating future work to decouple electrostatic gating from charge-transfer pathways, control ionic strength and specific adsorption, as well as functionalized graphene and other emerging 2D interfaces.

## Methods/Experimental

Sample: Graphene growth was done using a commercial BM Pro 4″ CVD reactor using 25 μm thick Cu foil (99.8%; Alfa Aesar), with specifics detailed in previous work,^16^ where the hydrogen annealing time was set to *t*_H2_ = 60 min, and the methane exposure time was set to *t*_CH4_ = 60 min. This gave a continuous film over the Cu catalyst with an average graphene domain size of approximately 100 μm. Float-zone silicon (FZ Si) substrates for the measurements were prepared by lithographically patterning Au markers onto the surface representing the 1 cm × 1 cm area of interest. Graphene transfer onto the FZ Si substrates was done by first drop-casting poly­(methyl methacrylate) (PMMA) (950 A4, diluted 1:4 in anisole) onto the Cu foil followed by etching any graphene on the back of the Cu foil with an O_2_ reactive ion etching at 50 W for 1 min. The Cu/graphene/PMMA was subsequently floated on 0.1 M aqueous ammonium persulfate for 6 h to remove the Cu, with the graphene/PMMA film moved into 3 subsequent DI water baths (each for 5 min). This graphene/PMMA was then transferred to the FZ Si, dried at room temperature in a cleanroom, baked at 110 °C on a hot plate for 10 min, and submerged in acetone for 12 h. Finally, the acetone was removed with an IPA rinse followed by drying with a continuous stream of N_2_.

Raman spectroscopy was performed after transfer using a 532 nm laser excitation with an exposure time of 1s and laser power in the order of 1 mW through a 20× objective, measured with a grating of 1800 l/mm in a Renishaw inVia Raman system. The resulting spatially resolved maps had their spectral fitted with Lorentzian functions at the locations of the D, G and 2D peaks.^19,33^

Liquid cell: A commercially available cell (Harrick Scientific TFC-S25–3) was customized for the experiments, which holds viewing windows with a diameter of 25 mm. The cell is used in an open configuration with just a single window. To prevent excessive liquid evaporation, the open volume is loosely covered by a lid. Around 2 mL of buffer solution with pH values of 4.01, 7, 9.22, 10.01, and 11.00 (Mettler Toledo) was used to immerse the graphene films. In between the different pH solutions during mapping, the liquid cell was rinsed with DI water to ensure a clean exchange of the respective buffer solution. For *in-operando* measurements, pH solutions were administered by a pipet and extracted by a peristaltic pump. The entire buffer exchange process took less than 10 s for each pH step.

THz-TDS: Reflection THz-TDS experiments were conducted using a TeraFlash system (Toptica, Munich, Germany) under nitrogen purge. In the setup, generated THz pulses are collected and collimated by a parabolic mirror with a focal length 101.16 mm where a 5 mm thick high resistivity silicon wafer is used to split-up a portion of the emitted THz pulses for focusing into the liquid cell at normal incidence by another parabolic mirror. The back-reflected THz pulse is then guided by the silicon beam splitter for detection via a third parabolic mirror. Such a reflection system achieves a THz spot size of approximately 1.5 mm at 1 THz. The use of a self-referenced measurement geometry *i.e*. first (air-substrate) and second (substrate-graphene-liquids) reflections as reference and sample signals, respectively, mitigates any potential phase misalignment errors in reflection THz-TDS.^52^ Using plane-wave approximation and for normal angle of incidence in free-space, eq 1 is derived for extracting graphene’s complex conductivity *σ̃* in an arbitrary medium of complex refractive index *n*_med_ *+ jk*_med_, which can be fitted using complex double Debye model^53,54^

$$\mathrm{\sigma ̃}=\frac{{(n}_{med}-n_{s}+2An_{med}\cos⌀+A^{2}\left(n_{med}{+n}_{s}\right))-j\left(k_{med}+A^{2}k_{med}+2Ak_{med}\cos⌀+2An_{s}\sin⌀\right)}{z_{0}\left(1+A^{2}+2A\cos⌀\right)}$$ 1

When the surrounding medium of graphene is free-space *i.e*. *n*_med_ = 1, *k*_med_ = 0, the analysis equation becomes

2 $$\mathrm{\sigma ̃}=\frac{{(n}_{s}\left(A^{2}-1\right)+A^{2}+2A\cos⌀+1)-j\left(2An_{s}\sin⌀\right)}{z_{0}\left(1+A^{2}+2A\cos⌀\right)}$$

as per previous work,^50^ where $A\;e^{j⌀}=\frac{\mathrm{r̃}\rho_{\mathrm{as}}\exp\left(\frac{j2n_{s}\omega l}{c}\right)}{\tau_{\mathrm{as}}\tau_{\mathrm{sa}}}$ with *r̃* being the Fourier transformed ratio between the reflected wave’s complex electric field from the sample to the reference of refractive index *n*_s_ and thickness *l*. Other parameters such as ρ_as_, τ_as_ and τ_sa_ correspond to Fresnel’s reflection ρ and transmission τ coefficients where the subscripts indicate the dielectric interfaces: Air (a), substrate (s). ω is the angular frequency, *c* is the speed of light in vacuum and *z*_0_ is the vacuum impedance (376.7 Ω). The use of plane-wave approximation is valid in eq 1 because of a relatively low numerical aperture (∼0.25). Graphene’s extracted AC conductivity spectra is then fitted using complex Drude model in eq 3 to extract DC conductivity σ_DC_ and scattering time *T* where realistic variations in substrate thickness can be corrected by iteratively multiplying a phase shift term $\exp\left(\frac{j2n_{s}\omega \Delta x}{c}\right)$ , where Δ*x* corresponds to thickness variation.^50^ Examples of fitted measurements are shown in SI. Using the relations in eqs 4 and 5, carrier drift mobility μ and density *N* can be extracted where *v*_F_ is the Fermi velocity at 10^6^ m/s.^47^

3 $$\mathrm{\sigma ̃}=\frac{\sigma_{\mathrm{DC}}}{1-j\omega T}$$

4 $$N=\frac{\pi \hbar^{2}}{q^{4}v_{F}^{2}}\frac{\sigma_{\mathrm{DC}}^{2}}{T^{2}}$$

5 $$\mu =\frac{qv_{F}}{\hbar \sqrt{\pi }}$$

Measurements of the samples were acquired at a step size of 500 μm where 200 waveforms were averaged to represent a data for a single pixel. A spatially averaged map of graphene’s conductivity (see Figure 2) can be generated by raster scanning the sample over an area of ∼20 × 20 mm^2^ inclusive of the viewing window without graphene, which took approximately 7 h and performing the analysis using eq 1 thereafter. The THz conductivity maps and Raman data were combined using image registration techniques. Key points on each map were identified and matched. These key points were then used to find a perspective transform using the random sample consensus (RANSAC) method, which provided a mapping function enabling the spatial association of the Raman spectra with the THz derived conductivity measurements, similar to prior work on ellipsometric and Raman data.^3,36^ Example alignment is shown in SI Figure S1. After data association the data was refined (with *e.g*. graphene free areas or areas overlapping the Au markers removed from the data) based on fitted characteristics from the Raman spectra (G and 2D peak heights, positions, and widths) and conductivity values. An example valid data map after this process is shown in SI Figure S2. To account for the THz spot size on the order of 1 mm, the Raman data used in this work was further refined using a Gaussian kernel of the same size, with the final maps representing the mean value of valid (*i.e*. not discarded) surrounding data points.

## Supplementary Material



## Data Availability

Additional data sets related to this publication are available from https://wrap.warwick.ac.uk/202368/.

## Notes

Detailed methodological information, per-sample statistics, Raman spectroscopy data, and THz responses (PDF)
