# Supplementary material for: High-Throughput In-Operando Terahertz Time-Domain Spectroscopy of Chemical Vapor Deposited Graphene for pH Sensing
Source: ACS Nano. 2026 Oct 21;20(43):28835–43. doi: 10.1021/acsnano.6c03948 (PMC13738731; doi:10.1021/acsnano.6c03948)
Supplement: Supplementary file 1 [file nn6c03948_si_001.pdf]

Supplementary Information for: High-Throughput In-Operando Terahertz Time-Domain Spectroscopy of Chemical Vapour Deposited Graphene for pH Sensing

Authors: Oliver J. Burton[1]*, Sebastian Engelbrecht[2], Thomas Goepfert[2], Ibtisam Hammad Abbasi[1], Linyuan Xin[1], Bernd M. Fischer[2], Hannah J. Joyce[1], Stephan Hofmann[1], Hungyen Lin[3]*.

[1] Department of Engineering, University of Cambridge, Cambridge CB3 0FA, United Kingdom

[2] French-German Research Institute of Saint-Louis, 5 rue du Général Cassagnou, 68301 Saint-Louis Cedex, France

[3] School of Engineering, University of Warwick, Coventry CV4 7AL, United Kingdom

* ob303@cam.ac.uk

* hungyen.lin@warwick.ac.uk

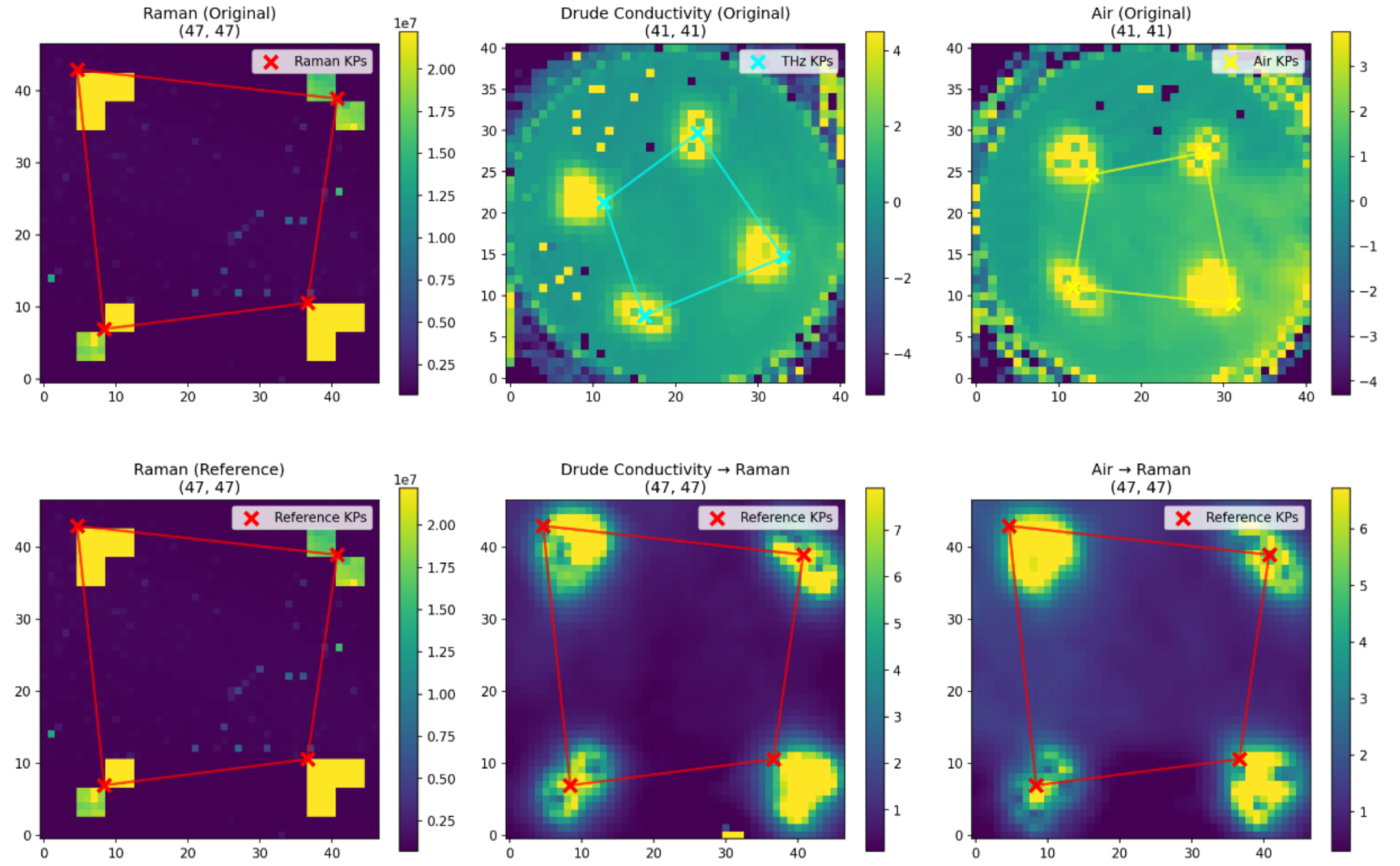

SI Figure S1: An example array of the alignment process for the data, whereby key points were identified for each data-type, followed by a geometric image transformation with a nearest neighbour interpolation in order to preserve the original data (i.e. true as measured values) for later comparison. Here we used patterned Au alignment markers, spaced 10 mm apart (centre to centre) as the key points for alignment, shown in these images as bright yellow spots. The x- and y- axes denote pixel position (with a pixel size of approximately 0.3 mm). The raman datatype is the aggregate intensity, whilst the drude conductivity and N_2 are both conductivity maps of the sample in water and N_2 environments respectively.

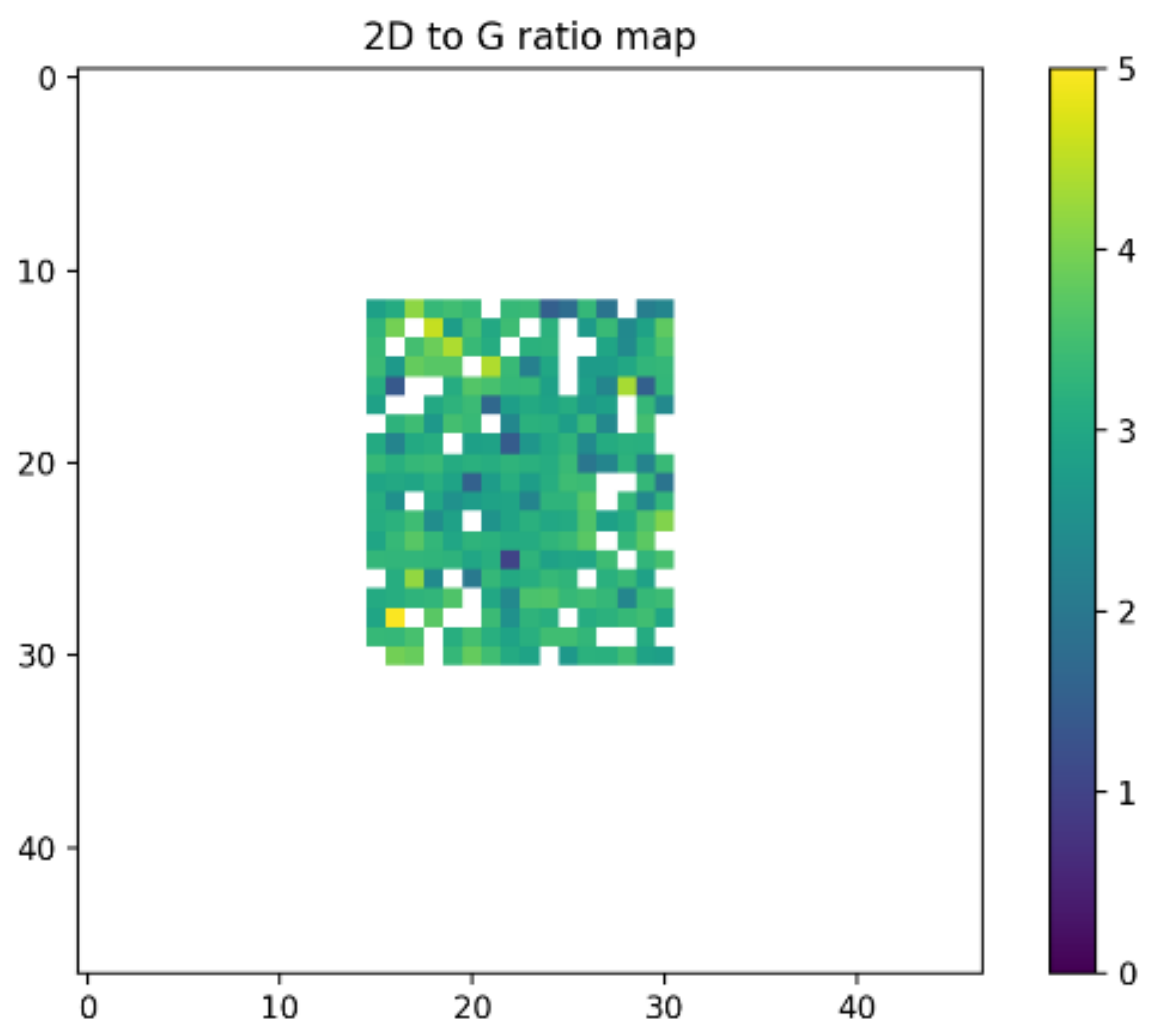

SI Figure S2: An example mask used on the spatially defined datasets used in this work. This shows all valid points after erroneous points were removed, *i.e.* points that showed no graphene in either the Raman analysis or the THz results, identified by no measurable conductivity, or no measurable peaks in the respective Raman spectra. A region of interest was also used to confine data to within a central region within the alignment markers to prevent erroneous outliers present (e.g. the Au markers used for alignment). The x- and y- axes denote pixel position (with a pixel size of approximately 0.3 mm).

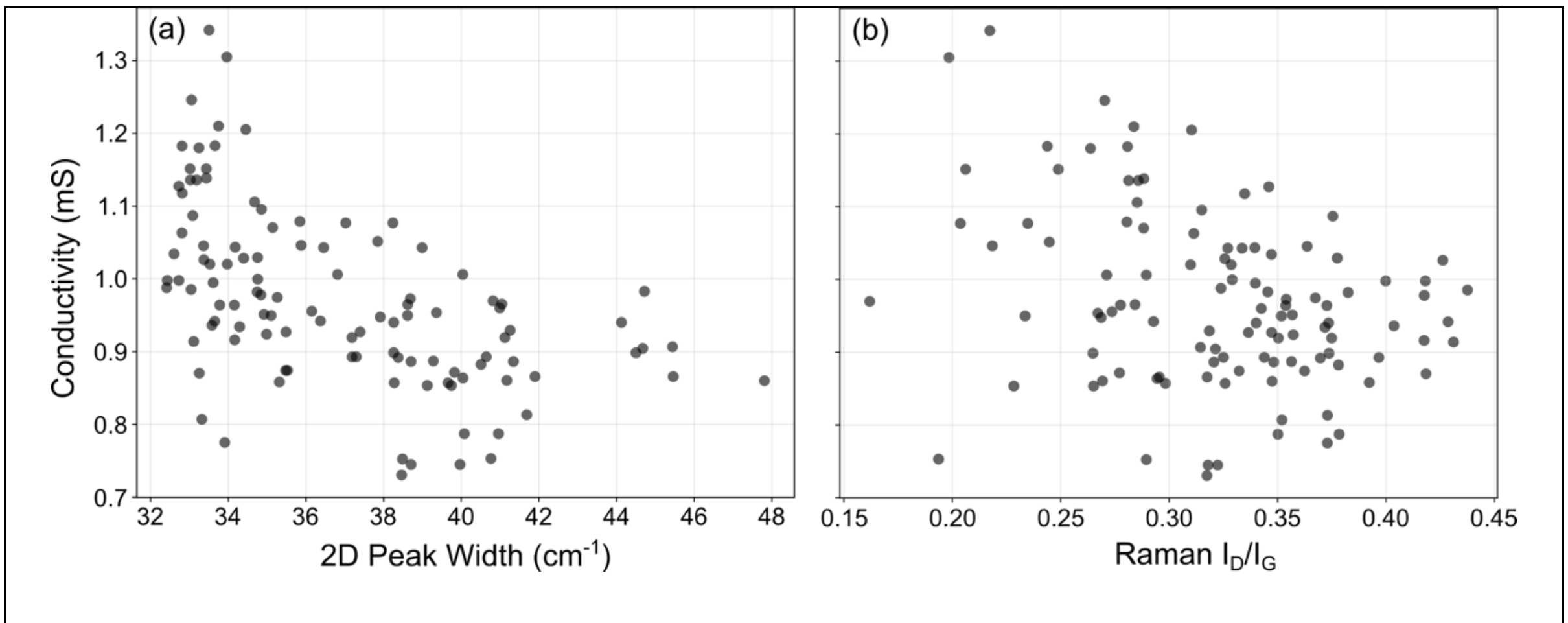

SI Figure S3: Scatter diagrams showing the correlation between THz measured conductivity and (a) the Raman 2D peak width and (b) the I_D/I_G ratio, showing that broadly speaking there is a negative correlation, with regions with wider 2D peak widths and higher D to G ratios yield lower extracted THz conductivities.

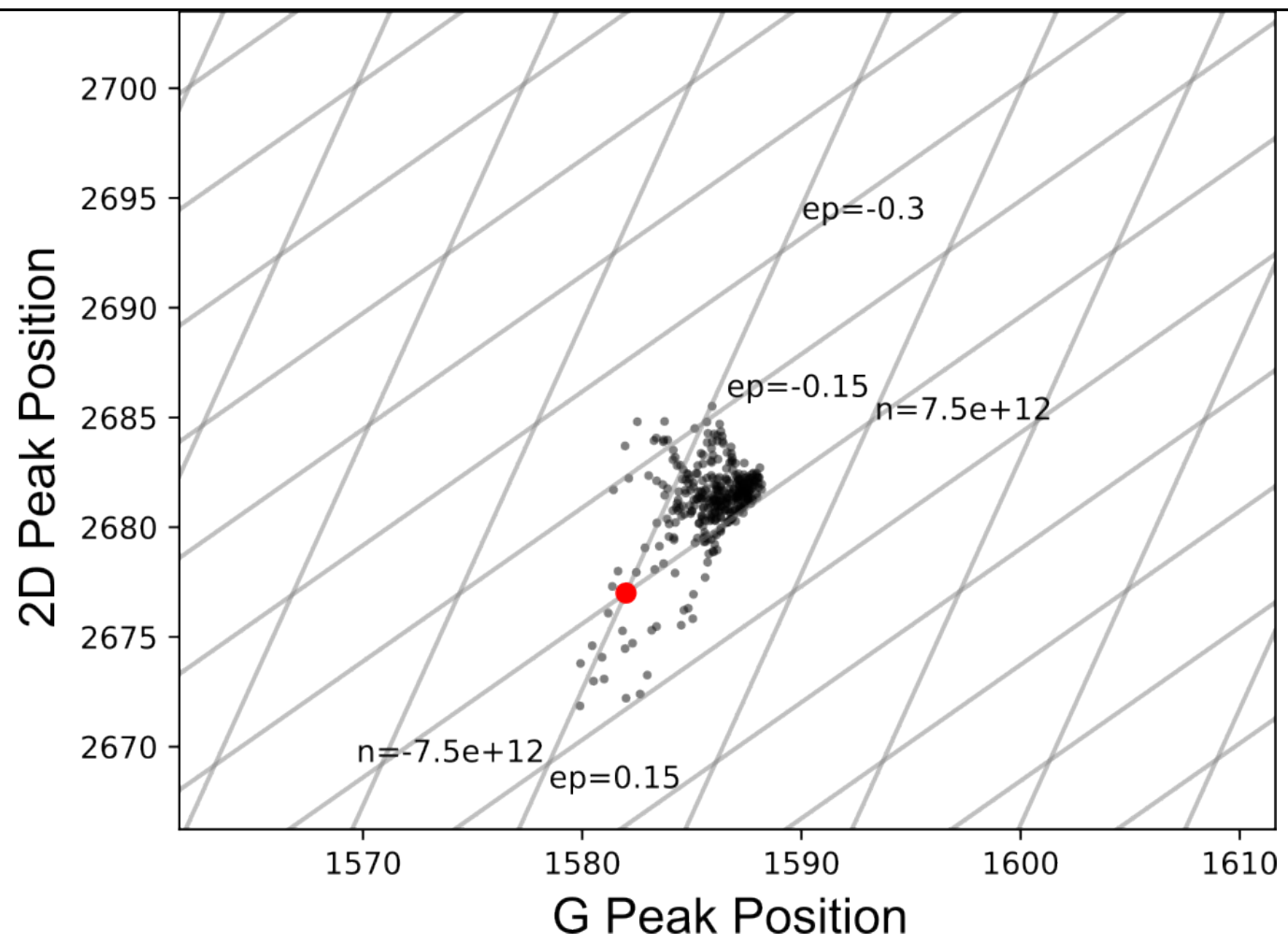

SI Figure S4: The Raman extracted doping and strain distribution (taken in air at ambient lab conditions) prior to liquid immersion and subsequent pH experiments. It should be noted that larger values of n indicate increased hole doping, based on the optical separation of strain and doping empirically derived in the literature.[1]

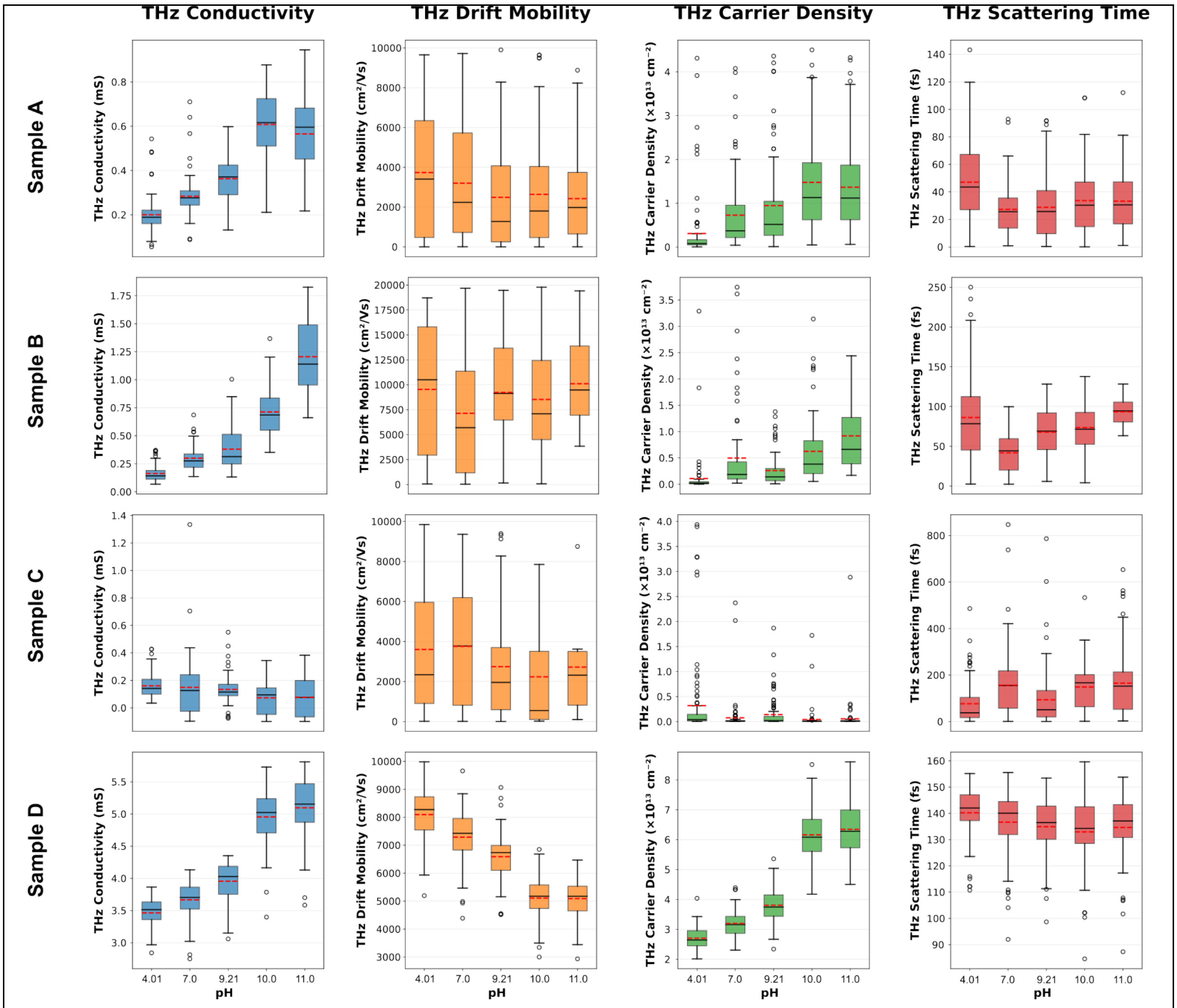

SI Figure S5: Presentations of aggregated extracted data from mapped samples A-D showing extracted THz conductivity, mobility, carrier density and scattering time of the carriers as a function of pH. Each box plot shows the range of accepted data as whiskers, the 25th and 75th quartiles, the median as black lines and the mean as the dashed red line. Note that Sample C had a significantly higher defect density, and as a result the conductivity is very low and shows no trend with changing pH, whereas A,B and C all show the same approximate increasing conductivity and carrier density with pH.

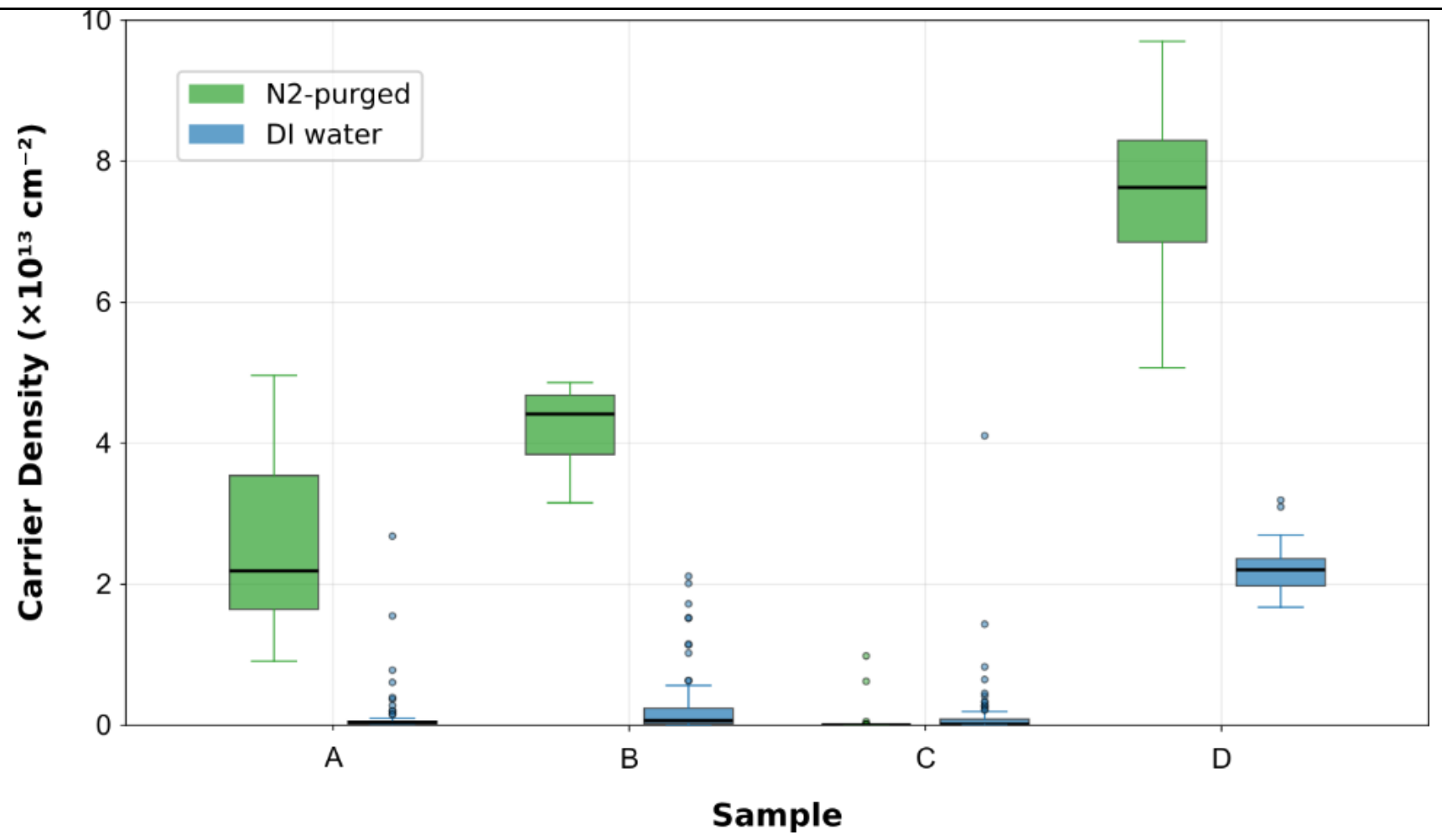

SI Figure S6: The THz extracted carrier density in N_2 prior to submersion in DI water, and whilst submerged in DI water (prior to varying pH for measurements) for the 4 spatially mapped samples, illustrating a significant decrease in carrier density, and implying a net change from p-doped to closer to neutral.

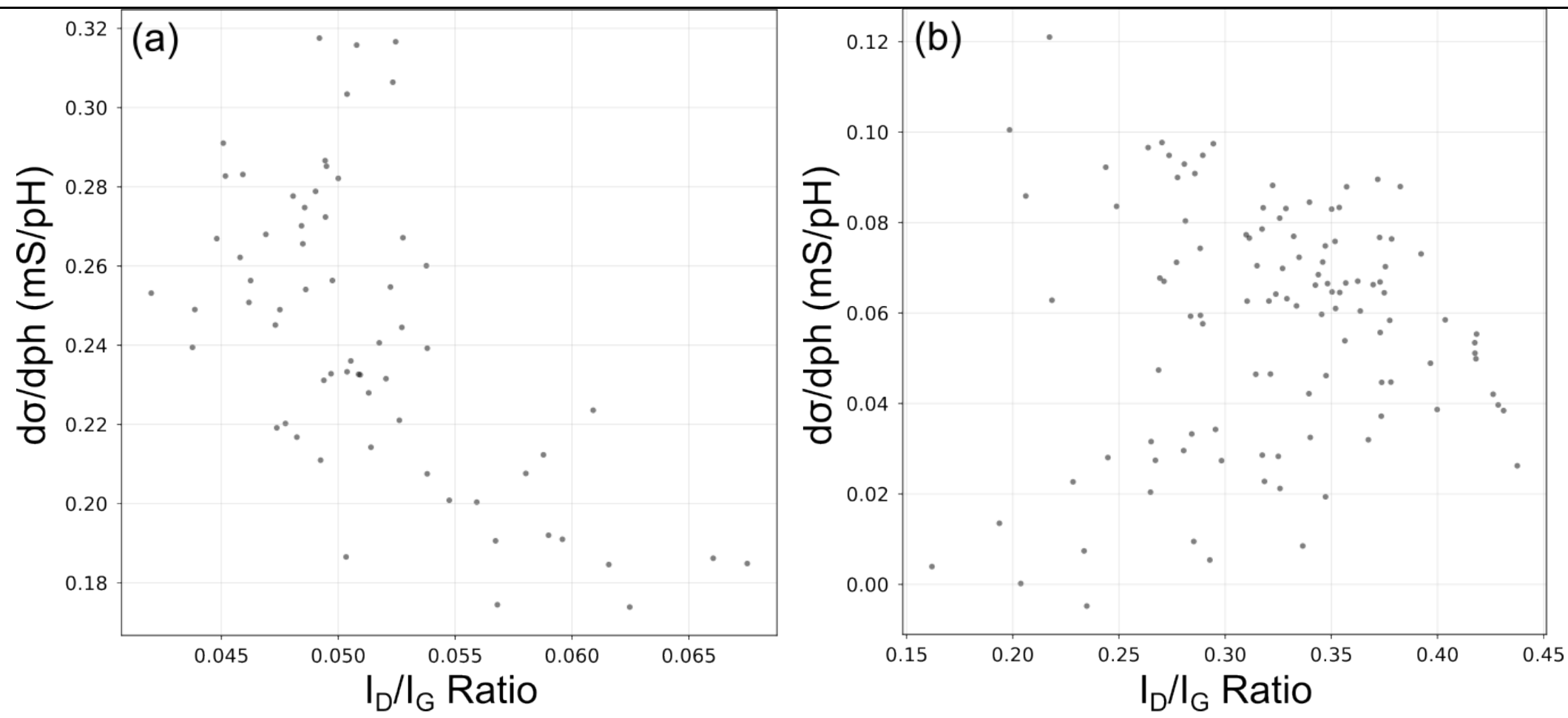

SI Figure S7: Plots of the pixelwise derivative of conductivity with respect to pH (*i.e.* the sensitivity) against the Raman I_D/I_G ratio for (a) a low defect sample (D; mean $I_D/I_G \sim 0.05$) and (b) a defective sample (A; mean $I_D/I_G \sim 0.35$).

Figure S7 shows the sensitivity of two samples as a function of Raman I_D/I_G ratios. It illustrates two broad regimes: in (a), the less defective graphene, an increase in I_D/I_G is correlated with steeply decreasing sensitivity. In the more defective sample shown in (b), however, we see two initial trends, where increasing I_D/I_G can either increase or decrease the sensitivity of a measurement, which might be indicative of the complexity of the system and representative of competing mechanisms of conductivity modulation. Previous literature on r-GO suggests that increasing defect density reduces sensitivity until $I_D/I_G \sim 0.35$, at which point the conductivity-pH slope inverts and sensitivity increases.[2] Whilst we do not see such inversion at $I_D/I_G \sim 0.35$, we do see a similar trend with CVD graphene, *i.e.* higher defect densities leads to a decrease of the average sensitivity of samples.

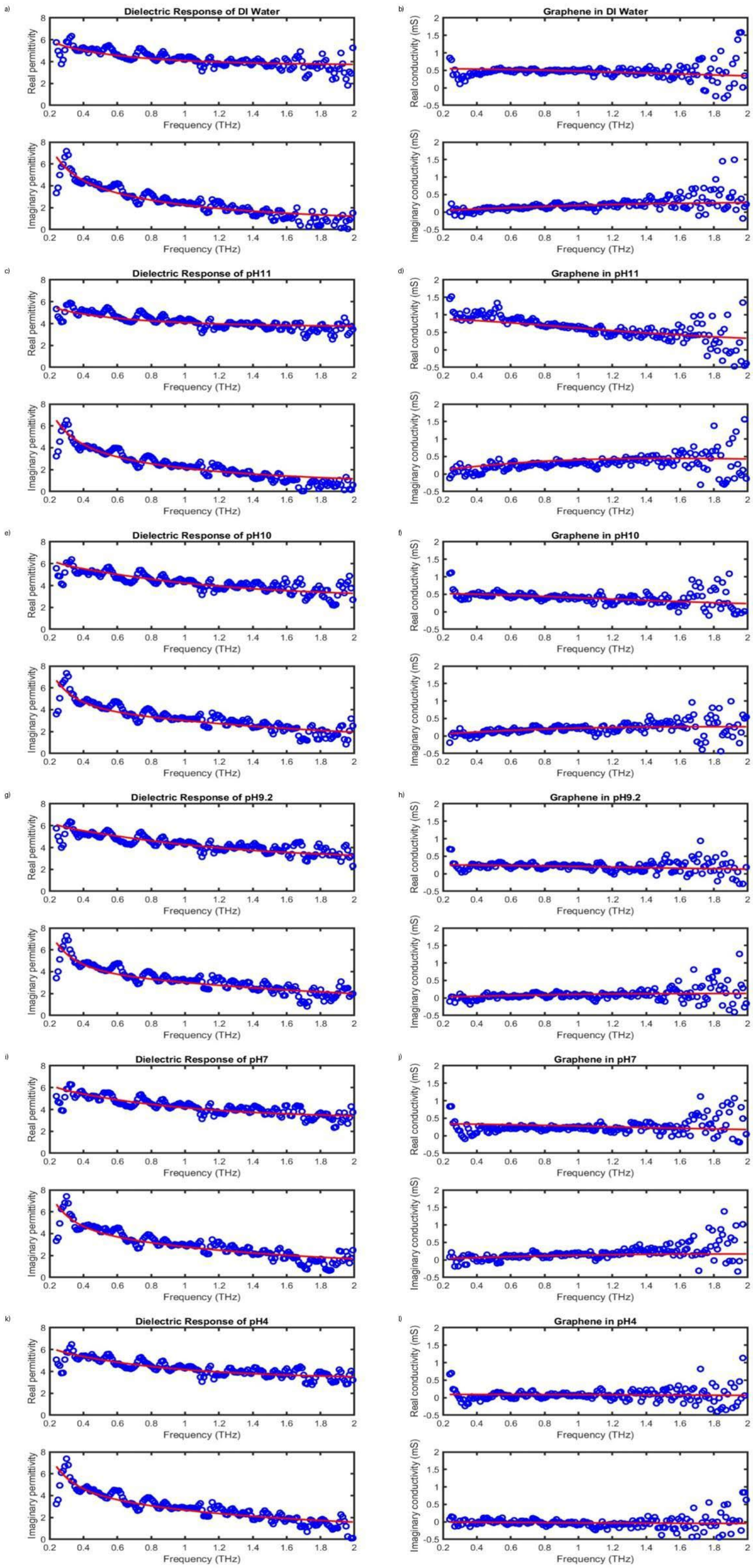

a)
Dielectric Response of DI Water
b)
Graphene in DI Water
c)
Dielectric Response of pH11
d)
Graphene in pH11
e)
Dielectric Response of pH10
f)
Graphene in pH10
g)
Dielectric Response of pH9.2
h)
Graphene in pH9.2
i)
Dielectric Response of pH7
j)
Graphene in pH7
k)
Dielectric Response of pH4
l)
Graphene in pH4
Real permittivity
Imaginary permittivity
Real conductivity (mS)
Imaginary conductivity (mS)
Frequency (THz)

SI Figure S8: (a, c, e, g, i, k) Measured and double Debye fitted complex dielectric response of the immersing medium with different pHs. (b, d, f, h, j, l) Measured and Drude fitted complex AC conductivity spectra of graphene on a representative pixel on sample B.
